# Characterization and phylogenetic analysis of the chloroplast genome in *Elaeocarpus duclouxii*

**DOI:** 10.1080/23802359.2024.2409759

**Published:** 2024-09-30

**Authors:** Zhitian Du, Haoliang Hu, Xingyu Zeng, Yongle Liu, Ying Chen, Zhuoyu Chenyang, Rong Zhu, Huang Kerui

**Affiliations:** Hunan Provincial Key Laboratory for Molecular Immunity Technology of Aquatic Animal Diseases, College of life and environmental sciences, Hunan University of Arts and Science, Changde, China

**Keywords:** *Elaeocarpus duclouxii*, chloroplast genome, phylogenetic analysis

## Abstract

*Elaeocarpus duclouxii*, an evergreen tree species, is renowned for its fruits rich in flavonoids exhibiting potent antioxidant properties. Despite its significance, the chloroplast genome of this plant has remained unexplored until now. Our study presents the first comprehensive sequencing and analysis of the *E. duclouxii* chloroplast genome, revealing a circular DNA molecule of 158,148 base pairs. This genome comprises a large single-copy region of 85,700 base pairs, a small single-copy region of 17,672 base pairs, and a pair of inverted repeat regions totaling 27,388 base pairs. The genome encodes 132 genes, including 87 protein-coding genes, 37 transfer RNA genes, and 8 ribosomal RNA genes. Phylogenetic analyses indicate a close evolutionary relationship between *E. duclouxii* and *E. sylvestris*. This study not only represents the first phylogenetic investigation of *E. duclouxii* but also establishes a crucial genomic foundation for future research area such as conservation genetics, evolutionary biology, and potential biotechnological applications.

## Introduction

*Elaeocarpus duclouxii* Gagnep 1910, a member of the Elaeocarpaceae family within the *Elaeocarpus* genus, is an evergreen tree indigenous to China’s evergreen forests. It flourishes at elevations between 700 and 950 m across several provinces, including Hunan, Yunnan, Sichuan, Guizhou, Guangxi, Guangdong, and Jiangxi. This species is characterized by pubescent young branches and leaves, with oblong, leathery foliage featuring 8–10 pairs of lateral veins. Its slightly pubescent petals are 10–12 lobed, and the tree bears 28–30 awnless stamens. The distinctive oval drupe, reaching up to 3 cm in length, possesses a notably furrowed endocarp (Wu et al. 2007). As part of the *Elaeocarpus* genus, *E. duclouxii* presents significant potential for ornamental and greening applications in garden landscapes. Its observed altitudinal preferences and habitat requirements suggest suitability for urban garden cultivation, though success hinges on adapting to local conditions and meeting the tree’s specific needs (Xie 2014).

*E. duclouxii’*s fruit flesh demonstrates versatility, being utilized in fruit juice and wine production, as well as consumed in dried form. Notably, flavonoid extracts from its fruits exhibit antioxidant properties in oils, positioning them as superior natural antioxidant sources (Jm et al. 2019). However, research specifically targeting *E. duclouxii* remains limited, with most available studies offering only general insights into the *Elaeocarpus* genus. The genetic information for this species is particularly scarce, resulting in an incomplete understanding of *E. duclouxii*, especially regarding its chloroplast genome.

This study aims to address these knowledge gaps by sequencing and characterizing the chloroplast genome of *Elaeocarpus duclouxii*. Additionally, it presents the first phylogenetic analysis of this species, laying a crucial foundation for future research into its evolutionary position and potential significance.

## Materials

**Figure 1.**
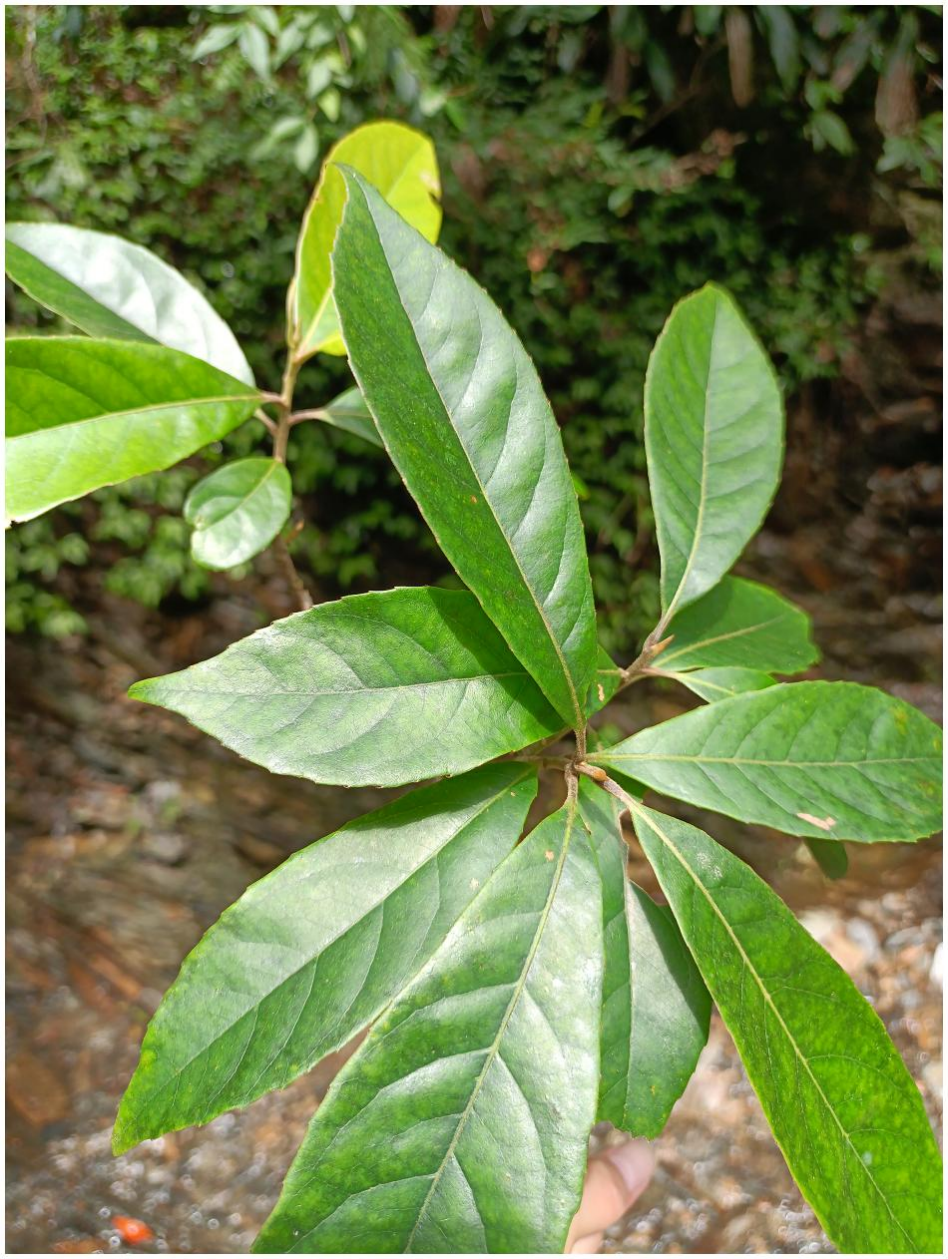
Picture of the collected sample of *E. duclouxii*. The picture was self-taken, samples were collected near the Hunan Nanyue Arboretum in Nanyue District, Hengyang City, Hunan Province, China, at an altitude of 335 m (27°16′43.70″N, 112°71′90.16″E). The leaves of *E. duclouxii* are clustered at the top of the branches, coriaceous, oblong, with a sudden sharp tip, wedge-shaped at the base, and are covered with brown villus below. The drupe is oval-shaped.

*E. duclouxii* plant specimens (Figure 1) were gathered in the vicinity of the Hunan Nanyue Arboretum, located in the Nanyue District of Hengyang City, Hunan Province, China, at an elevation of 335 meters (27°16′43.70″N, 112°71′90.16″E). The voucher specimen has been preserved in the Herbarium of Hunan University of Arts and Science and is available for consultation. (Contact: Ke Rui Huang, huangkerui008@163.com, Voucher Number HMDY001).

## Methods

The extraction and sequencing of the complete genomic DNA followed the protocol outlined by Zhang and colleagues (Zhang et al. 2023), genomic DNA was isolated from leaf samples preserved in liquid nitrogen using a commercial plant tissue DNA extraction kit (DN easy, TIANGEN Biotech Co., Ltd., Beijing). Subsequently, sequencing libraries were prepared and subjected to high-throughput sequencing on the Illumina HiSeq 2500 platform at Shanghai Personalbio Technology Co., Ltd., China. After using fastp (Chen et al. 2018) for quality control to eliminate inferior reads, a total of 62,035,020 reads were discarded. The chloroplast genome of *Elaeocarpus duclouxii* was then assembled de novo using GetOrganelle v1.7.5 (Jin et al. 2020). For the annotation of the chloroplast genome, we employed CPGAVAS2 (Shi et al. 2019), and the genome map was generated and visualized using CPGView (http://www.1kmpg.cn/cpgview/). For the phylogenetic analysis, we accessed 57 chloroplast genomes most closely related to *E. duclouxii* from GenBank. A total of 58 universal protein-coding genes were identified across all genomes. Each gene was individually aligned using MAFFT v7.313 (Rozewicki et al. 2019), refined with Gblocks 0.9b, and then merged into a supermatrix for each species, employing the maximum-likelihood method (Guo et al. 2022). Phylogenetic trees were derived using IQ-TREE v1.6.8 (Nguyen et al. 2015), applying the GTR+F + I + G4 model and supported by 5000 ultrafast bootstrap replicates.

## Results

**Figure 2.**
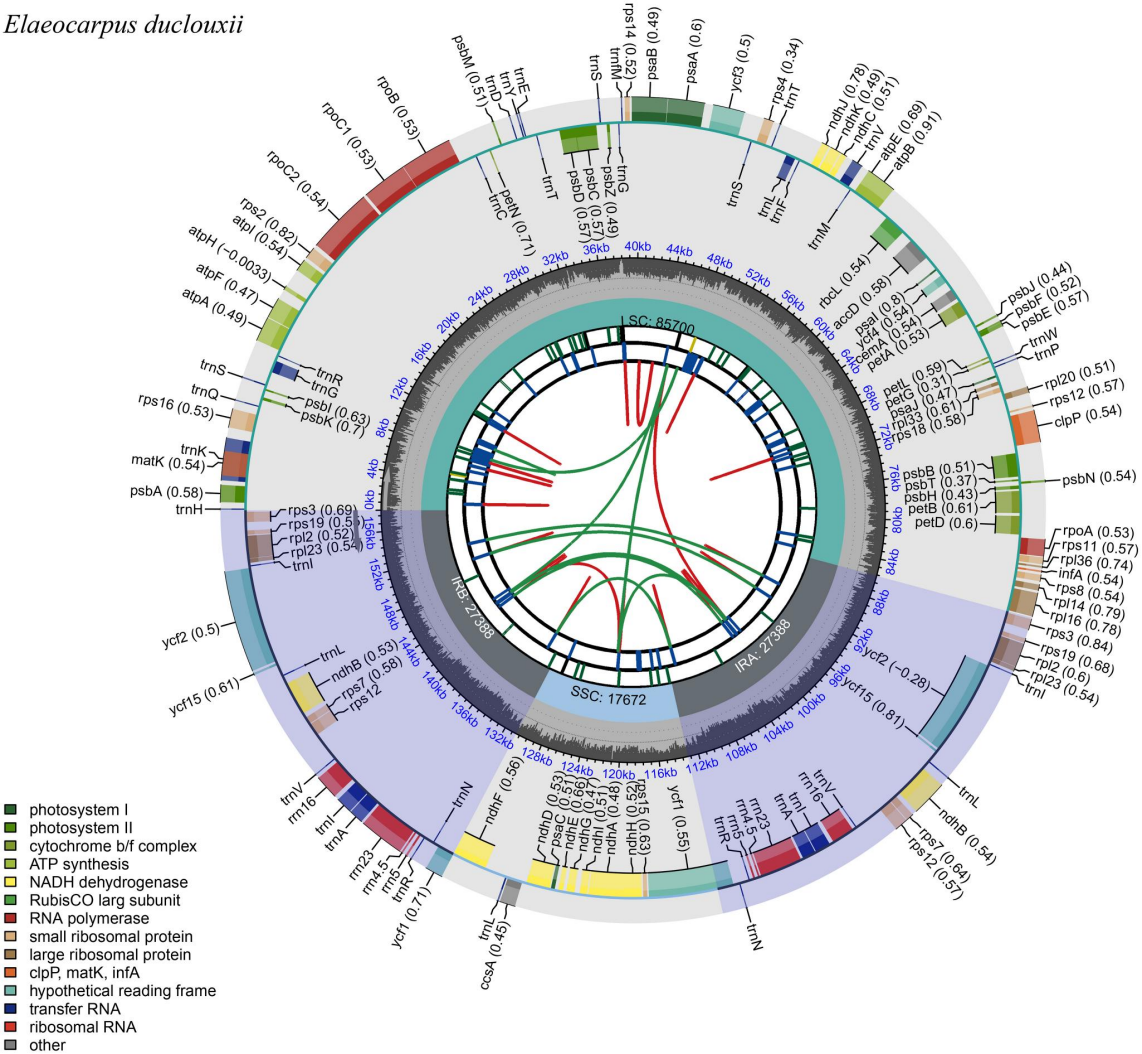
Gene map of the *Elaeocarpus duclouxii* chloroplast genome. From the center outward, the first track indicates the dispersed repeats; the second track shows the long tandem repeats as short blue bars; the third track shows the short tandem repeats or microsatellite sequences as short bars with different colors; the fourth track shows small single-copy (SSC), inverted repeat (ira and irb), and large single-copy (LSC) regions. The GC content along the genome is plotted on the fifth track; the genes are shown on the sixth track.

The chloroplast genome of *E. duclouxii* consists of a circular DNA molecule, 158,148 base pairs in length. It comprises a large single-copy (LSC) segment of 85, 700 base pairs, a small single-copy (SSC) segment of 17,672 base pairs, and a pair of inverted repeats (IR), each 27,388 base pairs long (illustrated in Figure 2 and Figure S1). In the entire chloroplast genome, the content of guanine plus cytosine (G + C) amounts to 37.06%. Broken down, the G + C content for the LSC, SSC, and IR regions equates to 34.97%, 31.16%, and 42.25%, respectively. The genome encodes 132 genes, comprising 87 protein-coding genes, 37 transfer RNA genes, and 8 ribosomal RNA genes, with 21 of these genes duplicated within the IR regions. The relationship between sequencing depth and local GC content is shown in Figure S2. In general, the trends of GC content and sequencing depth are consistent, indicating a positive correlation. This suggests that regions with higher GC content, which tend to have greater DNA stability, may be more efficiently amplified and sequenced. However, despite this correlation, our sequencing depth did not exhibit drastic fluctuations with changes in GC content, suggesting that our sequencing data is reliable and not overly biased by GC content variations. The structure of the cis-splicing genes and trans-splicing genes is depicted in Figure S3.

**Figure 3.**
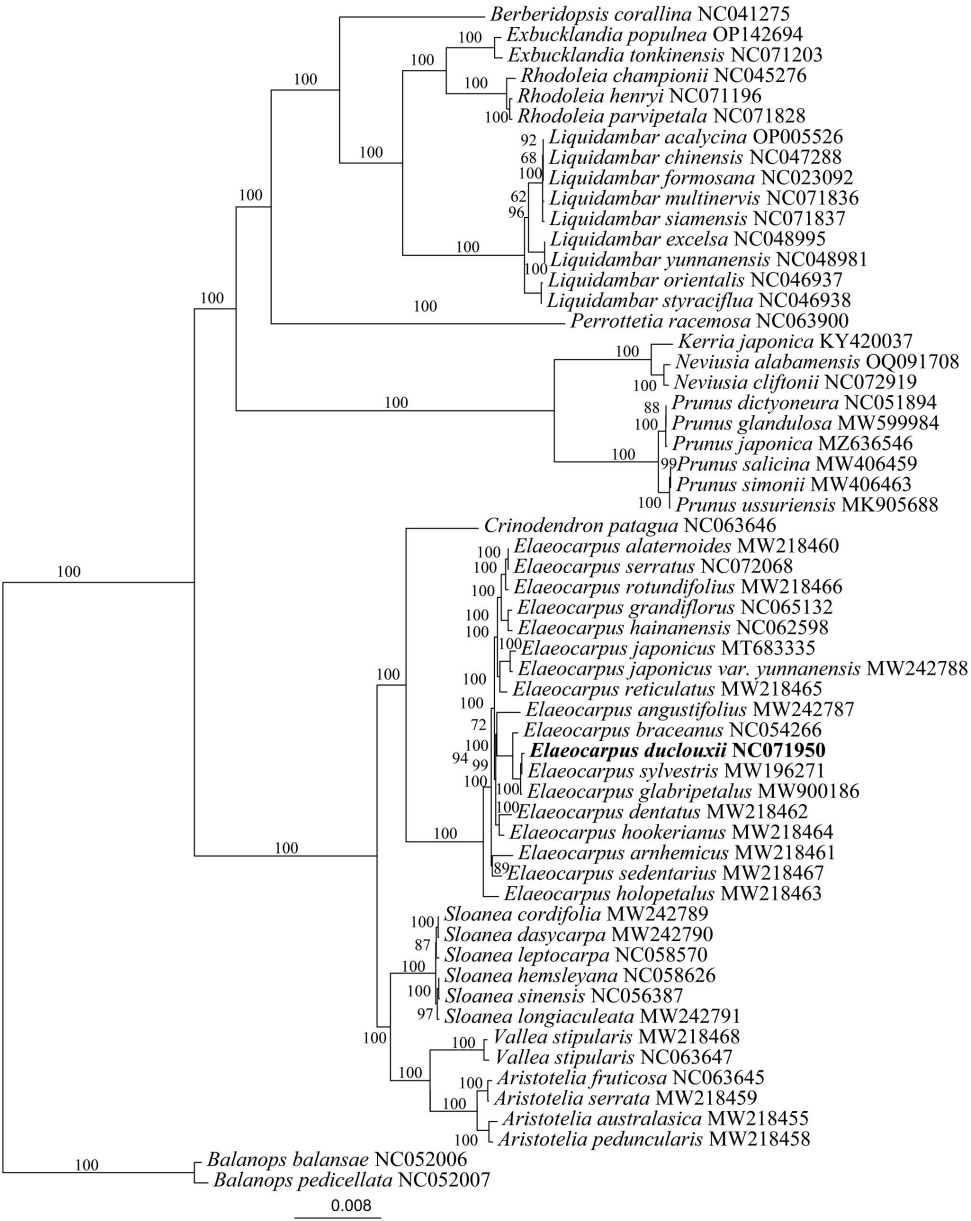
A maximum-likelihood tree of Elaeocarpus duclouxii and 57 related species was reconstructed by using the IQ-tree based on 58 protein-coding genes shared by all genomes. Bootstrap values are shown next to the nodes. The following sequences were used: *Aristotelia australasica* MW218455 (Maurin et al. 2022), *rhodoleia championii* NC045276 (Li et al. 2019), *Liquidambar acalycina* OP005526 (Lai et al. 2020), *Liquidambar chinensis* NC047288 (Wang et al. 2022), *Liquidambar formosana* NC023092 (Shi and Qin 2020), *Liquidambar orientalis* NC046937 (Or 2007), *kerria japonica* KY420037 (Luo et al. 2021), *Prunus glandulosa* MW599984 (Yi et al. 2021), *Prunus japonica* MZ636546 (Mu et al. 2021), *Prunus salicina* MW406459 (Fang et al. 2021), *Prunus simonii* MW406463 (Xu et al. 2021), *Elaeocarpus braceanus* NC054266 (Wang et al. 2020), *Elaeocarpus japonicus* MT683335 (Wang et al. 2021), *Elaeocarpus serratus* NC072068 (Sumanarathne et al. 2020), *Sloanea hemsleyana* NC058626 (Yang et al. 2022), *Sloanea leptocarpa* NC058570 (Sun et al. 2022), *Sloanea sinensis* NC056387 (Weng et al. 2021).

The phylogenetic analysis performed in this study identified a close relationship between *E. duclouxii* and *E. sylvestris*, supported by a 99% confidence level (Figure 3). This marks the first dedicated phylogenetic investigation of *E. duclouxii*, as prior studies did not specifically address this species. Additionally, the study included phylogenetic analyses of other species within the same genus as *E. duclouxii*. This study confirmed that *E. alaternaides* shares a close relationship with *E. rotundifolius*, and *E. hookerianus* with *E. dentatus*, corroborating previous research (Phoon 2015). However, *E. angustifolius* was not identified as closely related to *E. dentatus*, a discrepancy possibly due to the limited sample size and the paucity of data available for phylogenetic analysis, leading to inconsistencies with previous findings.

## Discussion

This study presents the first comprehensive analysis of the *Elaeocarpus duclouxii* chloroplast genome and its phylogenetic position. The genome’s structure and content align with typical angiosperm patterns, providing a valuable reference for comparative studies within the Elaeocarpaceae family. Our phylogenetic analysis reveals a close relationship between *E. duclouxii* and *E. sylvestris*, offering new insights into the evolutionary history of these species. While our findings largely corroborate previous research on other *Elaeocarpus* species relationships (Phoon 2015), the unexpected positioning of *E. angustifolius* highlights the complexities in resolving phylogenetic relationships within this genus. This discrepancy underscores the need for more extensive sampling and diverse data types in future studies.

The limited availability of *Elaeocarpus* chloroplast genome data in public databases remains a constraint, emphasizing the importance of continued sequencing efforts. Our contribution partially addresses this gap and provides a foundation for developing molecular markers for various applications, including population genetics and conservation studies. The characterization of the *E. duclouxii* chloroplast genome opens new avenues for research into chloroplast genome evolution within the Elaeocarpaceae family and could inform taxonomic classifications.

## Conclusion

This study significantly advances our understanding of *Elaeocarpus duclouxii* and its position within the *Elaeocarpus* genus by providing the first complete chloroplast genome sequence and phylogenetic analysis. The close relationship identified between *E. duclouxii* and *E. sylvestris* presents opportunities for further investigation into their shared evolutionary history and potential ecological similarities. The genomic resources and phylogenetic insights generated here have important implications for future research and applications, including the development of molecular markers and potential utilization in horticulture and traditional medicine.

## Supplementary Material

Supplemental Material.docx

## Data Availability

The complete chloroplast genome sequence of *Elaeocarpus duclouxii* has been submitted to the GenBank database. The accession number for this sequence is OP474143.1, which is automatically generated by the NCBI. In addition, the associated BioProject number is PRJNA1103375, the SRA accession number is SRR28776956, and the Bio-Sample number is SAMN41052082.
