# Supplementary material for: Characterization and phylogenetic analysis of the chloroplast genome in Elaeocarpus duclouxii
Source: Mitochondrial DNA B Resour. 2024 Sep 30;9(10):1307–12. doi: 10.1080/23802359.2024.2409759 (PMC11445928; doi:10.1080/23802359.2024.2409759)
Supplement: Supplemental Material.docx [file TMDN_A_2409759_SM3370.docx]

**Supplemental Material**

**
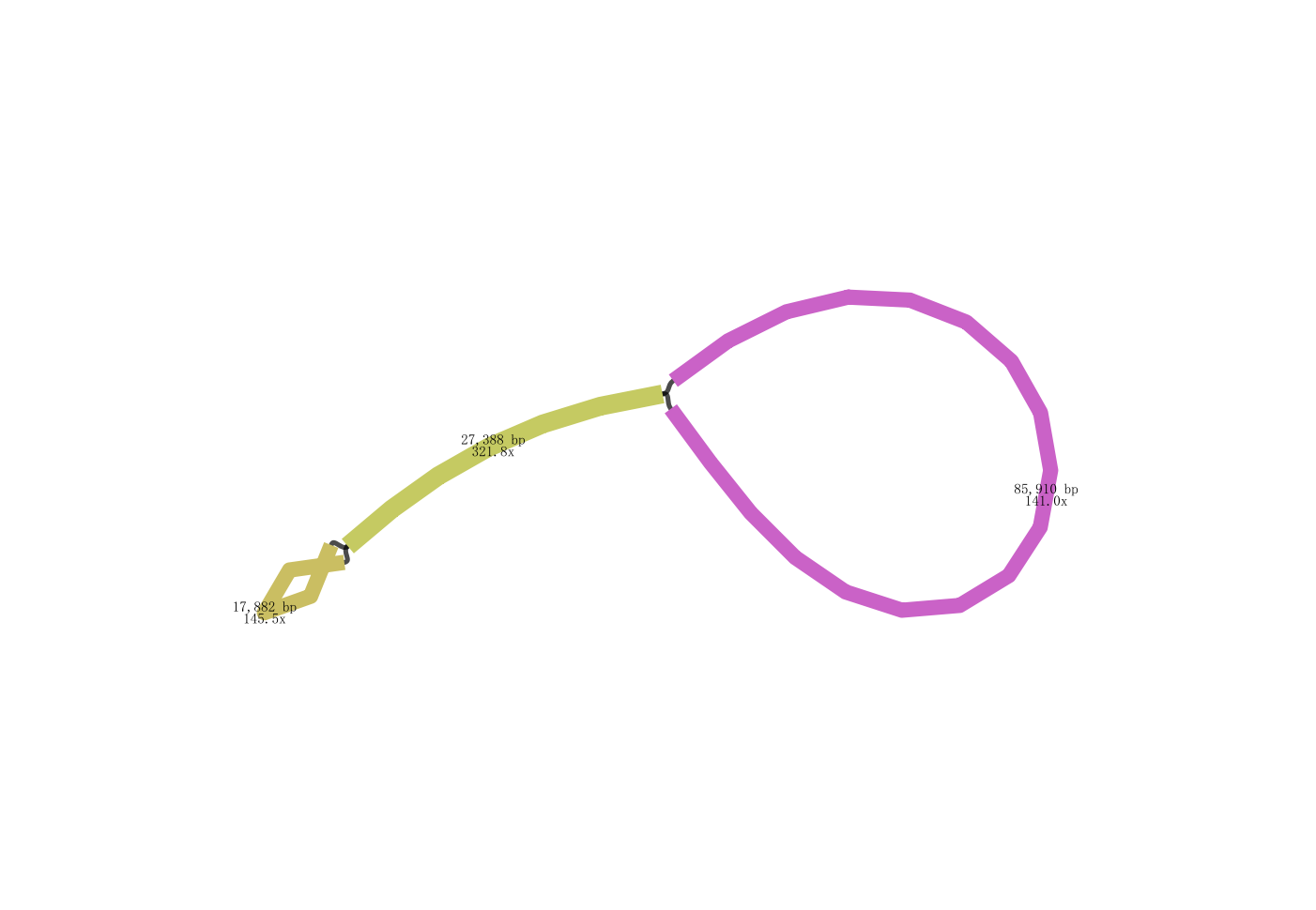
**

Figure S1. The schematic representation of the coverage depth for the entire chloroplast genome of *Elaeocarpus duclouxii* using Bandage. The numbers indicate the depths of different regions.

**
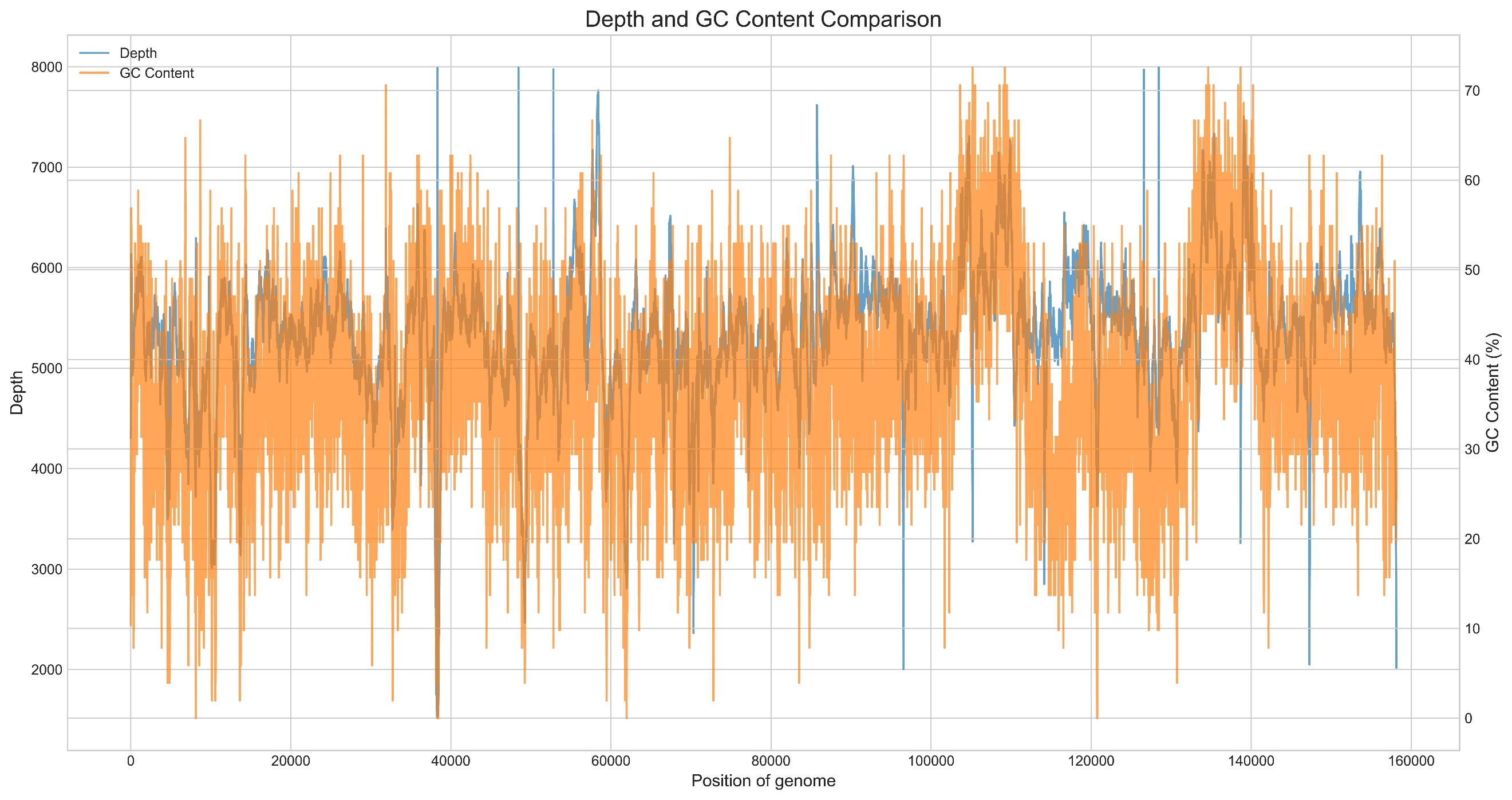
**

Figure S2. Comparison of chloroplast genome sequencing depth and GC content for *Elaeocarpus duclouxii*. The graph illustrates the distribution of chloroplast genome sequencing depth and GC content for the species *Elaeocarpus duclouxii*. The horizontal axis represents genomic position. The left vertical axis indicates sequencing depth, while the right vertical axis represents GC content. The blue line represents sequencing depth, and the orange line represents the local GC content around each position. GC content was calculated using a sliding window approach, where the GC content within a 25-base window flanking each position was determined.

**
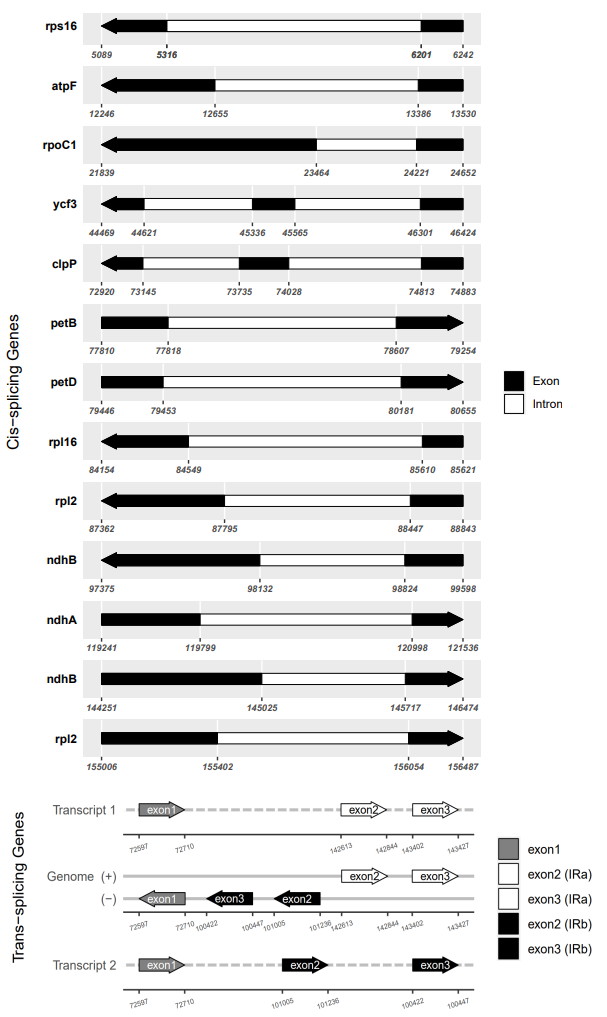
**

Figure S3. Schematic map of the cis-splicing genes and trans-splicing gene rps12 in the chloroplast genome of *Elaeocarpus duclouxii* using CPGView. The exons of the cis-splicing genes are shown in black; the introns are shown in white. The arrow indicates the sense direction of the gene. Please note that lengths of exons and introns are not drawn to scale.
